# Role of eRNAs in Cardiovascular Diseases

**DOI:** 10.2174/011573403X375542250529182602

**Published:** 2025-06-05

**Authors:** Yuqing Li, Chao Song, Jian Cui, XiangYu Fei, XiaoYong Lei, Huifang Tang

**Affiliations:** 1 School of Pharmacy, Hengyang Medical College, University of South China, 28 Western Changsheng Road, Hengyang, Hunan, 421001, China;; 2 Hunan Provincial Key Laboratory of Multi-omics and Artificial Intelligence of Cardiovascular Diseases, University of South China, Hengyang, Hunan, 421001, China;; 3 Department of Cardiology, The First Affiliated Hospital, Hengyang Medical School, University of South China, Hengyang, Hunan, 421001, China;; 4 Clinical Research Center for Myocardial Injury in Hunan Province, The First Affiliated Hospital, Hengyang, Hunan, 421001, China;; 5 Institute of Cardiovascular Disease, The First Affiliated Hospital, Hengyang Medical School, University of South China, Hengyang, Hunan, 421001, China

**Keywords:** Enhancer RNA, epigenetic, transcriptional regulation, cardiovascular disease, gene expression, IRENES

## Abstract

Enhancer RNAs (eRNAs), a class of non-coding RNAs transcribed from enhancer regions, have emerged as critical regulators of gene expression in cardiovascular diseases (CVDs), which are among the leading causes of morbidity and mortality in China. The pathogenesis of CVD is complex, involving precise regulation of diverse biological processes. Recent advances in epigenetics have highlighted the pivotal role of eRNAs in gene regulation. This review summarizes the fundamental characteristics of eRNAs and their mechanisms of action in CVD, focusing on how they regulate gene expression through enhancer-promoter looping, chromatin remodeling, and transcriptional control. Key eRNAs, including IRENES, CARMEN, LINC00607, HERNA1, PSMB8-AS1, and WISPER, are discussed in detail, emphasizing their roles in pathological processes, such as cardiac development, vascular remodeling, atherosclerosis, and fibrosis. These eRNAs interact with transcription factors and others to influence cardiovascular gene regulatory networks. Advances in high-throughput sequencing have identified eRNAs as potential biomarkers and therapeutic targets in CVDs, offering implications for diagnosis, treatment, and precision medicine. For instance, targeting CARMEN may attenuate atherosclerosis, while LEENE could address endothelial dysfunction. Despite their therapeutic potential, further studies are needed to elucidate the mechanisms underlying eRNAs function and their roles in CVD pathogenesis. A deeper understanding of eRNAs may pave the way for novel therapeutic strategies in cardiovascular medicine.

## 1 INTRODUCTION

Cardiovascular diseases (CVDs) are one of the leading causes of mortality worldwide, posing a significant threat to public health [1]. CVDs encompass a wide range of conditions, including coronary heart disease (CHD), hypertension, cardiac hypertrophy, and heart failure (HF), imposing a substantial burden on individual health and global healthcare systems. The etiology of cardiovascular diseases is complex and involves multiple biological mechanisms. Pathological processes, such as autophagy and apoptosis, have been shown to play a significant role in the onset of cardiovascular diseases across various species [2-5]. Therefore, there is an urgent need to develop new and effective targeted therapies for more precise risk stratification and disease management, which requires an in-depth understanding of the underlying molecular mechanisms that drive the progression of CVDs [6-8].

In recent years, with the advancement of genomic research, the importance of precise gene expression regulation in cardiovascular function has become increasingly evident. Epigenetics has provided novel insights into understanding these regulatory mechanisms, and a growing body of evidence suggests that epigenetic modifications play a critical role in the pathogenesis of many cardiovascular diseases [9, 10], particularly in how gene expression is regulated through DNA regulatory elements. These elements, including promoters, enhancers, silencers, and insulators, are central to gene expression control. Among them, enhancers have garnered significant attention. First described in 1981, the enhancer was cloned from the simian virus 40 (SV40) and consists of a 72 base-pair (bp) DNA sequence that increases the expression of associated genes in a cis-regulatory manner, independent of distance and orientation [11, 12]. Enhancers are critical for maintaining cardiovascular development and function [13, 14], and variations in enhancer sequences can lead to abnormal heart development [15, 16]. For example, the second heart field enhancer influences the cardiac gene regulatory network involving GATA6 and NKX2-5, affecting conotruncal defects caused by outflow tract malformations [17]. Cardiac development is governed by complex gene regulatory networks, known as cardiac gene regulatory networks (GRNs), which orchestrate the spatial and temporal regulation of intricate biological processes. Enhancers support these gene regulatory networks, enabling precise spatiotemporal patterns of gene expression in response to developmental and environmental stimuli [18]. It was not until 2010 that research on enhancers became a focal point. When researchers found that RNA polymerase II could aggregate at enhancer elements and was able to respond dynamically to signaling, and through the use of high-throughput sequencing, it was discovered that enhancers are widely transcribed into a type of non-coding RNA transcript called enhancer RNA [19, 20].

In recent years, there has been increasing attention on the study of enhancer RNAs (eRNAs) due to their unique biological properties and functions, particularly in gene expression regulation [21-23]. eRNAs function primarily by interacting with RNA polymerase II, transcription factors, and RNA-binding protein [24-26], facilitating the formation of enhancer-promoter loops [27], which, in turn, activate the expression of target genes. Additionally, eRNAs enhance chromatin accessibility by altering the spatial conformation of chromatin, thereby promoting the binding of RNA polymerase II to specific genomic loci [28]. Notably, eRNAs can also be synthesized before the transcription of target genes and interact with the negative elongation factor complex, thereby facilitating productive transcriptional elongation [29]. Research has shown that eRNAs play a critical role in cell-specific gene expression, particularly under pathological conditions. Abnormal eRNA expression has been closely linked to the dysregulated activation of disease-related genes in conditions, such as cancer, immune disease, and cardiovascular diseases [30-32]. As a result, eRNAs hold great promise as potential diagnostic and therapeutic targets for human diseases.

As eRNAs continue to be investigated across various fields, their roles in the heart remain largely unexplored. This review summarizes the fundamental characteristics and biological functions of eRNAs, with a particular focus on their molecular mechanisms and functions in the context of cardiovascular diseases. A deeper understanding of these mechanisms will enhance our knowledge of regulatory networks and highlight the potential clinical applications of eRNAs as biomarkers for cardiovascular diseases.

### 1.1 Search Strategy

We performed a comprehensive literature search in the PubMed database using the keyword “enhancer RNA” combined with “cardiovascular disease” or its related terms (*e.g.*, “atherosclerosis”, “heart failure”) from database inception to December 2024. Studies were included if they directly investigated the role of enhancer RNAs (eRNAs) in the pathogenesis, diagnosis, or treatment of cardiovascular diseases, covering experimental models (*in vitro*, *in vivo*, or clinical studies) and mechanistic explorations of eRNA functions.

## 2 BASIC CHARACTERISTICS AND BIOLOGICAL FUNCTIONS OF ERNAS

eRNAs are most simply defined as non-coding transcripts produced within a valid enhancer region. eRNAs can be generated as non-polyadenylated, bidirectional, and unstable transcripts [19, 33, 34]. They can also be produced as longer, unidirectional, polyadenylated, relatively stable, and sometimes spliced transcripts, which are often referred to as enhancer-associated long non-coding RNAs (lncRNAs) [20, 35-38]. Regions producing eRNAs are typically enriched with specific epigenetic marks, such as a high ratio of H3K4me1 (monomethylation) to H3K4me3 (trimethylation) and H3K27ac (acetylation) marks [34, 39, 40]. These marks are closely associated with enhancer activity, indicating that these regions are in an open chromatin state, which facilitates transcription factor binding. As most eRNAs are primarily non-polyadenylated and unstable, they are prone to rapid degradation. Consequently, they are mainly localized within the nucleus and enriched in chromatin-associated fractions [19, 20, 33, 41]. eRNAs do not encode proteins, and their structural characteristics suggest that eRNAs play a unique and crucial role in gene regulation.

**Fig. (1):**
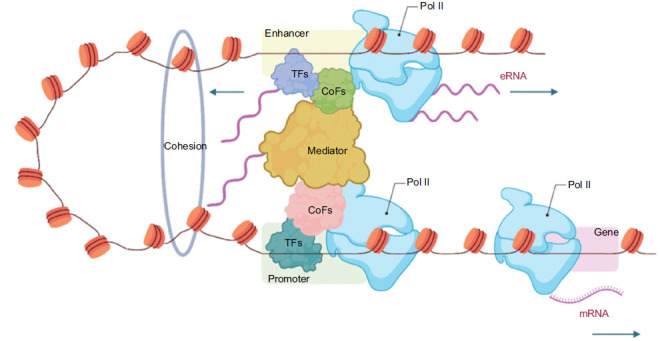
The enhancer and promoter form a loop structure, within which high levels of eRNA are expressed. eRNA interacts with the cohesin complex and the mediator complex to establish and stabilize the enhancer-promoter loop.

In addition, there is a more active type of enhancer known as a super-enhancer, which is similarly enriched with high levels of RNA polymerase II (RNA Pol II) [19] (Fig. **1**). Transcription factors associated with super-enhancers exhibit higher conservation across different species, with a conservation rate twice that of typical enhancer sites [42]. Super-enhancers surpass regular enhancers in terms of transcriptional activity, genomic coverage, transcription factor concentration, cell specificity, sensitivity to perturbations, and disease relevance, demonstrating stronger gene regulatory capabilities and potential therapeutic applications [43-46]. Super-enhancers can produce super-enhancer RNAs (seRNAs), most of which are unidirectionally polyadenylated, rendering them more stable and with a longer half-life compared to non-polyadenylated eRNAs [47]. seRNAs can regulate transcription in both cis and trans manners to coordinate precise patterns of gene expression [42, 46, 48]. In summary, super-enhancers play a crucial role in gene regulation and have significant potential for developing treatments for various diseases by influencing gene expression in specific ways, which could lead to new therapeutic approaches.

eRNAs were originally considered to be transcriptional noise enhancers [49]. Studies have shown that eRNAs are rapidly degraded by cells due to their short length, which supports the “noise hypothesis”. Indeed, most eRNAs are unstable, and their identification relies on various high-sensitivity sequencing and analytical techniques [50]. Precision Nuclear Run-On Sequencing (PRO-seq) [51] and Global Run-On Sequencing (GRO-seq) [52] are crucial methods for identifying eRNAs. Chromatin Immunoprecipitation Sequencing (ChIP-seq) [53], Cap Analysis of Gene Expression Sequencing (CAGE-seq) [54], and Transient Transcriptome sequencing (TT-seq) [55, 56] are also widely used in eRNA research. Additionally, the BruUV-seq method employs ultraviolet light to introduce transcription-blocking DNA damage, followed by bromouridine labeling of nascent RNA and subsequent deep sequencing to identify and enhance eRNA signal peaks [57]. Another high-sensitivity method for detecting unstable eRNAs is the adaptive CAGE approach (NET-CAGE), which involves lysing and isolating nascent RNA and using cap-trapping technology for 5' end sequencing [58]. Furthermore, the CRISPR/Cas9 system can be utilized to insert or delete DNA fragments at loci to study the role of eRNAs in gene regulation and cellular processes [59]. Fluorescence *in situ* hybridization (FISH) can also be employed with specific probes to detect eRNAs, confirming their presence and colocalization [60]. Advanced imaging protocols for single-molecule fluorescence *in situ* hybridization (smFISH) allow for the visualization and localization of RNA molecules within cells [61, 62]. These methods provide comprehensive, rich tools for studying eRNAs, advancing our understanding of enhancer RNA functions and their roles in gene regulation.

The multifaceted functions of eRNAs in gene regulation have become a significant focus of research in recent years. Studies have shown that enhancers and promoters form loop structures within which high levels of eRNAs are expressed. eRNAs interact with the cohesin complex and the mediator complex to stabilize these structures and modulate the expression of target genes [63, 64] (Fig. **1**). In estrogen-dependent transcriptional activation, eRNAs enhance the strength of enhancer-promoter loops, contributing to gene activation [65]. Moreover, eRNA plays a pivotal role in chromatin remodeling and gene regulation. It acts as a chromatin modifier, regulating chromatin remodeling in various genomic regulatory regions to facilitate gene activation and dosage regulation [28, 66]. Mechanistically, eRNA interacts with chromatin-modifying enzymes, such as CBP/p300, leading to an increase in H3K27 acetylation and a decrease in H3K4 trimethylation, thereby promoting gene transcription. This chromatin modification pattern favors a more open chromatin conformation, facilitating gene expression. Furthermore, these modifications offer potential therapeutic strategies for CBP/p300-associated diseases, including cancer and neurodegenerative disorders [67]. This modification renders chromatin more active, facilitating gene expression. Additionally, eRNAs are involved in the formation of chromatin loops, a process essential for gene regulation [68, 69]. eRNAs regulate the heparanase (HPSE) gene and drive the chromatin-modifying hnRNPU/p300/EGR1/HPSE axis in cancer, promoting cancer progression [70]. Secondly, eRNAs influence gene transcriptional activity by interacting with transcription factors or transcriptional regulators [68, 71, 72]. For example, the eRNA CARMEN binds to the transcription factor GATA, thereby activating the expression of cardiac-specific genes (such as NKX2.5 and MESP1) and maintaining the differentiation state and function of cardiac cells [73]. The eRNA of RGS1 recruits the transcription factor FOXJ3 to precisely activate RGS1 expression and further promotes osteoclast differentiation *via* the PLC-IP3R-dependent Ca^2+^ signaling pathway, accelerating the pathological progression of rheumatoid arthritis, leading to bone destruction and aggravated inflammation [25]. Moreover, eRNA can promote the recruitment of RNAPII and other transcription factors, enhancing the transcriptional activity of promoter regions [74]. It also facilitates the release of the NELF from the RNAPII complex, thereby increasing transcription elongation efficiency and precisely regulating gene expression [29]. Notably, the functions of eRNA are not limited to transcriptional regulation; it may also indirectly affect mRNA stability and translation efficiency through transcriptional activation of enhancer regions, thus participating in post-transcriptional regulation [75, 76]. These functions highlight the significant roles that eRNAs play in various aspects of gene regulation, from enhancer regulation to chromatin remodeling, transcription, and post-transcriptional regulation. Collectively, these findings underscore the critical role of eRNAs, as products of enhancers, in regulating gene expression through multiple mechanisms, emphasizing their indispensable position in gene expression regulation [27].

## 3 ERNAS IN CARDIOVASCULAR DISEASES

With the advent of new high-throughput genomics and epigenomics technologies, many researchers are now exploring novel frontiers of enhancer-associated long non-coding RNAs (lncRNAs) in cardiac development and disease. The mechanisms of several well-studied eRNAs involved in cardiovascular diseases, including IRENES, CARMEN, LINC00607, HERNA, PSMB8-AS1, and WISPER, are summarised in Table **1**. These factors function not only as eRNAs but also as cardiac enhancers and the expression of their target genes. Numerous studies have shown that the discovery of eRNAs mechanisms provides a promising therapeutic target for the treatment of cardiovascular diseases.

### 3.1 IRENES

IRENES (Intergenic Regulatory Element Nkx2-5 Enhancers) are lncRNAs transcribed from the enhancers of the cardiac GRN determinant transcription factor (TF) Nkx2-5. Nkx2-5 mutations have been recognized as a major cause of congenital heart defects (CHDs) [17]. Nkx2-5 is a gene that plays a critical role in heart development and cardiac homeostasis, with its expression being tightly regulated both temporally and spatially during carcinogenesis. The Nkx2-5 locus contains an enhancer region that encodes two eRNAs, namely IRENE-SS and IRENE-div. These two eRNAs have contrasting functions in regulating Nkx2-5 expression. IRENE-SS functions as a classical eRNA with transcriptional activator properties, recruiting NKX2-5 to its gene enhancer to promote Nkx2-5 transcription. Conversely, IRENE-div acts as a non-conventional eRNA with transcriptional repressor functions, enhancing enhancer acetylation and reducing SIRT1 binding to inhibit Nkx2-5 transcription. The balance between these two eRNAs is crucial for the regulation of Nkx2-5 expression and the normal development and homeostasis of cardiomyocytes [60]. Additionally, Nkx2-5 can transcriptionally regulate Tbx5/GLS2/ferroptosis in cardiomyocytes by directly binding to its super-enhancer and the lncRNA Snhg7, thereby enhancing their activation. This mechanism plays a significant role in cardiac hypertrophy [77].

### 3.2 CARMEN

CARMEN (cardiac mesoderm enhancer-associated noncoding RNA), also known as CARMEN, is a noncoding RNA associated with cardiac mesodermal enhancers, initially identified in studies of cardiac differentiation [73]. It has been implicated in both cardiac development and vascular biology. CARMEN, originally termed MIR143HG, serves as the host gene for miR-143 and miR-145, which are generated through alternative splicing. The miR-143/145 cluster primarily regulates smooth muscle cell (SMC) differentiation and phenotypic switching in response to vascular injury and remodeling [78]. In the context of atherosclerosis, CARMEN has been reported to drive VSMCs toward a pro-proliferative, pro-migratory, and dedifferentiated phenotype *via* the miR-143/145 axis *in vitro* [79]. In mouse models with CARMEN knockout induced by CRISPR-Cas9, the absence of CARMEN accelerated the progression of atherosclerosis, emphasizing its regulatory role. Furthermore, CARMEN was found to colocalize with the nuclei of VSMCs in both murine and human atherosclerotic plaques, and its deletion in low-density lipoprotein receptor-deficient (LDLR-/-) mice significantly reduced the size of atherosclerotic lesions. Mechanistically, CARMEN interacts with serum response factor (SRF) to regulate VSMC plasticity, thereby mediating the development of atherosclerosis [80]. Intriguingly, other studies suggest that CARMEN promotes the contractile phenotype of VSMCs *via* interaction with the transcriptional cofactor myocardin, independent of the miR-143/145 pathway. Notably, CARMEN deletion exacerbated vascular injury in mouse models, while its overexpression partially restored vascular function in a rat model of arterial injury [81].

Beyond atherosclerosis, CARMEN has been implicated in other cardiovascular pathologies. During cardiac development, CARMEN interacts with the core components of polycomb repressive complex 2 (PRC2), SUZ12, and EZH2 to regulate the differentiation of cardiac progenitor cells (CPCs) and cardiac specification [73]. In abdominal aortic aneurysms (AAAs), CARMEN prevents aneurysm formation by modulating SRF activity and inhibiting the phenotypic switching of VSMC [82]. Moreover, in hypertensive patients, CARMEN expression is significantly upregulated and positively correlated with left ventricular mass index [83]. In addition to this, one of the CARMEN isoforms, CARMEN-201, is essential for SMC formation. C-201 represses the expression of cardiomyocyte-associated transcription factors, including ISL1, IRX1, IRX5, and SFRP1, by interacting with the transcription factor REST. This prevents CPCs from developing into cardiomyocytes and instead promotes their differentiation into SMCs [84].CARMN has also been extensively studied in other areas, including colorectal cancer [85], cervical cancer [86], and breast cancer [87]. These findings underscore the importance of studying CARMEN as an enhancer RNA (eRNA), given its multifaceted roles across various systems and diseases. Its diverse functions provide promising avenues for exploring CARMEN as a potential biomarker and therapeutic target in the diagnosis and treatment of human diseases.

### 3.3 LINC00607

LINC00607, a long non-coding RNA (lncRNA) derived from a super-enhancer, has recently been identified and is enriched in cell types associated with atherosclerosis [88]. Predominantly expressed in endothelial cells (ECs) and VSMCs in human arteries [89], LINC00607 plays a significant role in regulating endothelial cell functions, such as angiogenesis, VEGF production, and wound healing, as well as VSMC functions, including cell proliferation and migration. Notably, c-Myc has been identified as an upstream transcriptional regulator of LINC00607, acting as a stimulus-dependent transcription factor that inhibits LINC00607 transcription under high glucose conditions. Consequently, LINC00607 may contribute to vascular dysfunction in conditions, such as diabetes, and inhibitors of LINC00607 can reverse the dysfunction induced by high glucose and TNFα in endothelial cells [89]. Further research is required to elucidate the molecular mechanisms through which LINC00607 regulates its downstream targets and its involvement in different cell types and environments.

LINC00607 expression levels are also associated with endothelial dysfunction and vascular inflammation. By interacting with the pro-inflammatory and pro-fibrotic gene SERPINE1, LINC00607 plays a crucial role in regulating endothelial cell functions [90]. Additionally, LINC00607 is involved in endothelial dysfunction and endothelial-to-mesenchymal transition (EndoMT). It interacts with genes, such as SMAD3, COL4, and CTGF, which promote these processes. Furthermore, research has demonstrated that LINC00607 affects endothelial cell function by regulating gene expression and chromatin accessibility, thereby influencing angiogenesis and vascularization. Knockdown of LINC00607 results in the downregulation of several genes associated with the VEGF signaling pathway. LINC00607 maintains the transcription of ERG target genes by interacting with the chromatin remodeling factor BRG1, affecting chromatin accessibility and the availability of transcription factor binding sites, thus regulating gene transcription [91]. In summary, these studies suggest that LINC00607 may represent a potential therapeutic target for cardiovascular diseases.

### 3.4 LEENE

LEENE (lncRNA that enhances eNOS expression) is a recently discovered lncRNA involved in endothelial cell homeostasis, identified through its epigenetic regulation of vascular endothelium [92]. LEENE is differentially regulated by pulsatile shear stress and oscillatory shear stress, and its expression is upregulated by KLF2 and KLF4. LEENE enhances endothelial nitric oxide synthase (eNOS) transcription through chromatin interaction, thereby promoting nitric oxide production and modulating endothelial cell (EC) function [93]. In the development of atherosclerosis, LEENE alleviates the transcriptional inhibition of ABCA1 by promoting the degradation of Forkhead Box O1 (FoxO1), thereby enhancing cholesterol efflux, reducing lipid accumulation, and mitigating the progression of atherosclerosis [94]. Additionally, LEENE plays a crucial role in the pathophysiology of hypertension. In an AngII-induced hypertension model, LEENE-deficient mice exhibited more severe hypertension, cardiac hypertrophy, fibrosis, and kidney damage, suggesting that LEENE may be an important regulator of blood pressure. However, its exact mechanism of action requires further investigation [95].

Beyond its role in atherosclerosis and hypertension, LEENE also plays a pivotal regulatory role in peripheral artery disease and ischemic lesions related to diabetes. Research has shown that LEENE interacts with LEO1, a key component of the RNA polymerase II-associated factor complex, and the critical angiogenesis transcription factor MYC, promoting the transcription of pro-angiogenic genes KDR and NOS3, thus regulating angiogenesis and tissue perfusion [96]. Moreover, in high-glucose environments, LEENE expression is suppressed, leading to impaired angiogenic capacity. However, increased LEENE levels can enhance ischemic repair, providing a potential therapeutic target for ischemic diseases related to diabetes [97]. Although multiple studies have revealed the role of LEENE in cardiovascular diseases, its precise regulatory mechanisms and potential clinical applications still require further exploration.

### 3.5 HERNA1

HERNA1 (hypoxia-inducible enhancer RNA) represents a novel cardiac-specific non-coding RNA that is robustly induced under pathological stress. As an eRNA, HERNA1 plays a crucial role in modulating gene programs related to growth, metabolism, and contraction in disease contexts. HERNA1 is produced *via* the direct binding of hypoxia-inducible factor 1 alpha (HIF-1α) to hypoxia response elements within the enhancer-promoter region. This binding facilitates the induction of adjacent genes, including synaptotagmin XVII (Syt17) and SMG1, which are implicated in the hypoxic response. The interaction of HERNA1 with the promoter regions of Syt17 and Smg1 triggers hypoxia-responsive transcriptional activity, leading to pathological growth, glycolytic phenotype, and Syt17-driven contractile dysfunction, which are characteristic of pressure-overload cardiomyopathy. Studies have demonstrated that inhibition of HERNA1 post-disease onset can reverse left ventricular hypertrophy and dysfunction, thereby enhancing overall survival rates. Consequently, HERNA1 emerges as a potential molecular target for cardiovascular disease therapy [98, 99].

### 3.6 PSMB8-AS1

PSMB8-AS1 has garnered attention as an eRNA associated with various malignancies [100-102]. Emerging evidence also implicates its involvement in autoimmune diseases, such as systemic lupus erythematosus [103]. Recent findings have indicated that PSMB8-AS1 plays a crucial role in the progression of cardiovascular diseases. Notably, PSMB8-AS1 levels are significantly elevated in the serum of patients with coronary artery disease, with a marked increase in expression observed within atherosclerotic plaques. PSMB8-AS1 promotes the adhesion of monocytes and macrophages and accelerates vascular inflammation. Mechanistically, PSMB9 mediates PSMB8-AS1-induced expression of VCAM1 and ICAM1. Downregulation of PSMB8-AS1 or inhibition of the NONO/PSMB9/ZEB1 axis has been found to exert protective effects against atherosclerotic cardiovascular disease [104]. As a PSMB9 inhibitor, KZR-616 has entered phase III clinical trials for the treatment of lupus nephritis [105]. Developing novel enhancer-associated long non-coding RNA-based strategies to combat atherosclerotic cardiovascular diseases is of paramount importance.

### 3.7 WISPER

WISPER (Wisp2 super-enhancer–associated RNA) is a polyadenylated, multi-exon transcript enriched in cardiac fibroblasts (CFs). WISPER expression is upregulated in response to stress conditions, such as myocardial infarction (MI). It exerts its function through interaction with the RNA-binding protein TIAR (TIA1 cytotoxic granule-associated RNA-binding protein), leading to the stabilization of transcripts bound to TIAR, including the prolyl 4-hydroxylase 2 (Plod2) gene involved in collagen synthesis. Knockdown of WISPER in CFs results in decreased expression of Plod2 and other ECM-related genes, leading to reduced collagen production and fibrosis. Beyond its role in CFs, WISPER also acts as a sponge for microRNAs (miRNAs) targeting fibrosis-related genes. By sequestering these miRNAs, WISPER prevents their inhibitory effects on fibrosis-related genes, thereby further promoting fibrosis. In animal models, prophylactic depletion of WISPER using GapmeRs (antisense oligonucleotides) reduces cardiac fibrosis post-MI. Knockout of the WISPER gene results in decreased expression of ECM-related genes and improved cardiac function. In summary, WISPER plays a crucial role in regulating cardiac fibrosis through its interaction with TIAR, stabilization of ECM-related transcripts, and sequestration of fibrosis-associated miRNAs. Its depletion shows potential as a therapeutic strategy to reduce cardiac fibrosis and remodeling [106, 107].

## CONCLUSION

In recent years, with the advent of new high-throughput genomics and epigenomics technologies, there has been a growing interest in new frontiers of enhancer landscapes and associated lncRNAs in cardiac development and disease [108-110]. The pathogenesis of cardiovascular diseases is a complex biological process that is tightly regulated by multiple pathways. Dysregulation of these pathways is mediated by genetic and epigenetic changes in both protein-coding genes and non-coding regulatory elements, which have been shown to play roles in cardiovascular diseases [15, 16]. eRNAs are involved in the complex cardiac gene regulatory networks (GRNs) in the cardiovascular system, and comprehensive studies on eRNAs can reveal mechanisms of cardiovascular diseases. For example, the expression of eRNAs is associated with major regulatory factors in cardiac development, such as Mesp1, NKX2.5, and Gata4. Moreover, lncRNAs have the potential for reprogramming factors, enabling the direct reprogramming of non-cardiomyocytes, such as cardiac fibroblasts, into cardiomyocytes. eRNAs hold great potential in cardiac regeneration [60].

**Fig. (2):**
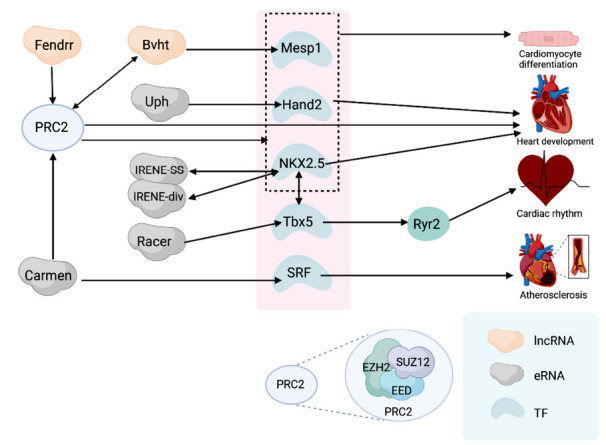
Enhancer RNAs (eRNAs) play a crucial role in the regulation of cardiac genes, forming a regulatory network that includes key transcription factor (TF) genes, such as Mesp1, Nkx2.5, Tbx5, Hand2, and SRF. Within this network, Bvht acts upstream by regulating Mesp1. Specifically, Bvht modulates SUZ12, a component of the Polycomb Repressive Complex 2 (PRC2), which is composed of SUZ12, EZH2, and EED. Through its interaction with SUZ12, Bvht further influences the promoter regions of cardiac-specific genes, such as Mesp1, Hand2, and Nkx2.5, playing a key role in cardiomyocyte differentiation. Fendrr is another molecule that interacts with the PRC2 components, EZH2 and SUZ12, which is critical for heart development. Additionally, the eRNA CARMEN interacts with PRC2 components SUZ12 and EZH2, contributing to cardiomyocyte differentiation. CARMEN also associates with SRF, playing a role in the regulation of atherosclerosis. The Tbx5-associated eRNA RACER, by binding to chromatin, may stabilize the connection between enhancers and promoters, directly contributing to the regulation of the calcium-handling gene Ryr2, thereby affecting cardiac rhythm. Nkx2.5 and its associated eRNAs, IRENE-SS and IRENE-div, are also crucial in the regulation of heart development. There is also molecular interaction between the key cardiac transcription factors NKX2.5 and TBX5. Together, these essential TFs and eRNAs form a regulatory network governing cardiac gene expression.

Beyond cardiac regeneration, the coordinated spatiotemporal execution of cardiac gene regulatory networks (GRNs) is crucial during heart development and the onset of CVDs. eRNAs interact with specific transcription factors (TFs) to participate in these GRNs and modulate the progression of CVD. Key TF genes, such as Mesp1, Nkx2.5, GATA4, TBX5, Hand2, and SRF, play essential roles in cardiac gene regulation. Within these networks, the long non-coding RNA (lncRNA), Bvht, has emerged as an important regulatory locus-transcribed lncRNA, previously identified to be enriched in cardiac-specific enhancers [111]. Bvht acts upstream in the transcriptional regulation of Mesp1 by modulating SUZ12, a component of Polycomb Repressive Complex 2 (PRC2). During cardiomyocyte differentiation, SUZ12 targets and binds to promoter regions of cardiac-specific genes, such as Mesp1, Hand2, and Nkx2-5. The binding of SUZ12 to these promoters is critical for regulating the epigenetic state and expression levels of these genes [32]. Numerous lncRNAs have been shown to interact with PRC2; one such transcript, Fendrr, essential for heart and body wall development in both humans and mice, interacts with the PRC2 components EZH2 and SUZ12. Fendrr facilitates the accumulation of PRC2 at target gene promoters (such as Foxf1 and Pitx2), thereby increasing H3K27me3 (a repressive mark) levels and inhibiting the expression of these genes [112]. Similarly, the eRNA CARMEN interacts with SUZ12 and EZH2, components of PRC [73]. Additionally, Hand2, a critical transcription factor for reprogramming fibroblasts into cardiomyocytes, is tightly regulated by upstream enhancers. The Hand2-associated long non-coding RNA, referred to as upper hand (Uph), functions by regulating the Hand2 gene expression during cardiac development [113]. Furthermore, Tbx5-associated eRNA RACER, by binding to chromatin, may stabilize the enhancer-promoter interactions and directly modulate the expression of the calcium-regulating gene Ryr2 [114]. Molecular interactions also exist between the cardiac transcription factors NKX2.5 and TBX5 [115]. These key TFs interact with eRNAs, forming a comprehensive cardiac gene regulatory network (Fig. **2**).

eRNAs also show potential clinical applications in the diagnosis and treatment of cardiovascular diseases (CVDs) [116]. For example, in Fabry disease (FD), which frequently affects the heart, the expression of enhancer RNAs can be monitored for diagnosis and therapeutic purposes [117]. In patients with atherosclerosis, the associated vascular inflammation may be altered by controlling eRNA expression, thereby improving prognosis [80]. Cardiac fibroblast-specific eRNA expression may be regulated to reduce fibrosis [107]. For example, endothelial nitric oxide synthase may play an important role in cardiovascular physiology by catalyzing the production of nitric oxide. eRNA molecules like LEENE could be used as therapeutic targets to control hypertensive disorders [96].

Therefore, research on these non-coding transcriptional regions is crucial for the treatment of cardiovascular diseases. Although the number of identified eRNAs is still limited, they have already shown great potential as new therapeutic strategies for cardiovascular diseases. Thus, it is necessary to further investigate the relationship between eRNAs and cardiovascular diseases. In the future, a deeper understanding of the mechanisms by which eRNAs regulate gene expression and their specific roles in the pathogenesis of diseases will help identify new therapeutic targets and biomarkers for cardiovascular diseases. Through these studies, eRNAs are expected to become an important component of precision medicine and personalized therapy, offering new breakthroughs in the prevention, diagnosis, and treatment of cardiovascular diseases.

## Figures and Tables

**Table 1:** The role of eRNAs in cardiovascular diseases.

**Identified eRNAs**	**Target Gene**	**Disease Type**	**Mechanism**	**References**
IRENES	Nkx2-5	CHD	IRENE-SS promotes Nkx2-5 transcription, while IRENE-div inhibits Nkx2-5 transcription by reducing SIRT1 binding.	[60]
CARMEN	miR-143, miR-145, SRF	AS, AAA, and HTN	CARMEN regulates the phenotype transformation of smooth muscle cells and related disease processes through interaction with transcription factors, such as myocardin and SRF.	[80, 82, 83]
LINC00607	c-Myc	AS and CVDs	LINC00607 regulates endothelial cell function and vascular formation through interactions with c-Myc and BRG1.	[88, 91]
LEENE	eNOS	HTN and CAD	LEENE regulates endothelial cell function and vascular formation by enhancing eNOS transcription and interacting with LEO1 and MYC.	[95, 96]
HERNA1	Syt17, SMG1	AS and HCM	HERNA1 regulates hypoxic responses and pathological gene expression by binding to the hypoxia-inducible factor HIF-1α and activating gene transcription within the enhancer-promoter region.	[98, 99]
PSMB8-AS1	PSMB 9	CVD	PSMB8-AS1 accelerates monocyte and macrophage adhesion and vascular inflammation by activating the NONO/PSMB9/ZEB1 axis to promote the expression of VCAM1 and ICAM1.	[104]
WISPER	Wisp2	AOS and CM	WISPER stabilizes the transcripts of collagen synthesis genes by interacting with the RNA-binding protein TIAR.	[106, 107]

## LIST OF ABBREVIATIONS

CHD: Coronary Heart Disease
CVDs: Cardiovascular Diseases
eRNAs: Enhancer RNAs
GRNs: Gene Regulatory Networks
HF: Heart Failure
lncRNAs: Long Non-coding RNAs

## DECLARATION

Some clipart icons appearing in Figures **1** and **2** were generated using BioRender (https://biorender.com).
